# Neonatal AAV delivery of alpha-synuclein induces pathology in the adult mouse brain

**DOI:** 10.1186/s40478-017-0455-3

**Published:** 2017-06-23

**Authors:** Marion Delenclos, Ayman H. Faroqi, Mei Yue, Aishe Kurti, Monica Castanedes-Casey, Linda Rousseau, Virginia Phillips, Dennis W. Dickson, John D. Fryer, Pamela J. McLean

**Affiliations:** 1 Department of Neuroscience, Mayo Clinic, 4500 San Pablo Rd, Jacksonville, FL 32224 USA; 2 Neurobiology of Disease Graduate Program, Mayo Clinic Graduate School of Biomedical Sciences, Jacksonville, FL USA

**Keywords:** Alpha-synuclein, Neonatal injection, Viral vector model, Aggregation

## Abstract

Abnormal accumulation of alpha-synuclein (αsyn) is a pathological hallmark of Lewy body related disorders such as Parkinson’s disease and Dementia with Lewy body disease. During the past two decades, a myriad of animal models have been developed to mimic pathological features of synucleinopathies by over-expressing human αsyn. Although different strategies have been used, most models have little or no reliable and predictive phenotype. Novel animal models are a valuable tool for understanding neuronal pathology and to facilitate development of new therapeutics for these diseases. Here, we report the development and characterization of a novel model in which mice rapidly express wild-type αsyn via somatic brain transgenesis mediated by adeno-associated virus (AAV). At 1, 3, and 6 months of age following intracerebroventricular (ICV) injection, mice were subjected to a battery of behavioral tests followed by pathological analyses of the brains. Remarkably, significant levels of αsyn expression are detected throughout the brain as early as 1 month old, including olfactory bulb, hippocampus, thalamic regions and midbrain. Immunostaining with a phospho-αsyn (pS129) specific antibody reveals abundant pS129 expression in specific regions. Also, pathologic αsyn is detected using the disease specific antibody 5G4. However, this model did not recapitulate behavioral phenotypes characteristic of rodent models of synucleinopathies. In fact no deficits in motor function or cognition were observed at 3 or 6 months of age. Taken together, these findings show that transduction of neonatal mouse with AAV-αsyn can successfully lead to rapid, whole brain transduction of wild-type human αsyn, but increased levels of wildtype αsyn do not induce behavior changes at an early time point (6 months), despite pathological changes in several neurons populations as early as 1 month.

**Electronic supplementary material:**

The online version of this article (doi:10.1186/s40478-017-0455-3) contains supplementary material, which is available to authorized users.

## Introduction

Parkinson’s disease (PD) is a progressive neurodegenerative disease characterized by a complex motor disorder known as Parkinsonism which manifests as resting tremor, bradykinesia, rigidity, and postural abnormalities. Additionally, there are many non-motor symptoms such as olfactory deficit, sleep abnormalities, depression, and cognitive disturbances that are increasingly recognized as being integral components of the disease [7, 12]. Pathologically, PD is characterized by a selective loss of dopaminergic neurons in the substantia nigra (SN) and abnormal intracellular protein deposits called Lewy bodies (LB) that are mainly composed of fibrillary alpha-synuclein (αsyn) protein aggregates. Large bodies of evidence point to αsyn involvement in PD, since mutations and multiplications of the gene encoding αsyn have been linked to onset of familial forms of PD [20, 40, 45] and aggregation of αsyn is causally linked to sporadic forms of the disease [48]. In recent years these pathological hallmarks have also been observed in multiple system atrophy and dementia with LBs (DLB), which together with PD are referred to as αsynucleinopathies [19, 47].

Modeling PD and its related disorders in animals to recreate specific pathogenic events and behavioral outcomes is a crucial step in basic research for mechanistic studies and therapeutic screening. The discovery of the involvement of αsyn in pathogenesis prompted the development of numerous animal models based on αsyn overexpression primarily through genetic manipulation methods in rodents [28] and later using viral vector technology that can specifically target a region of interest [22, 27]. To date, αsyn transgenic mice harboring various extents of pathological features of PD, decreased dopamine, and behavioral impairments but no significant dopaminergic cell loss [14, 30] have been developed. In contrast, viral vector-mediated overexpression of αsyn models display a progressive pathology associated with clear dopaminergic neurodegeneration, replicating primary motor symptoms of PD [50]. These models represent perhaps one of the most reproducible rodent models of PD, however, the targeted and local αsyn overexpression has its own limitations and these models do not possess non-motor symptoms observed in PD patients. αSyn overexpression studies have been useful in uncovering the relationship between αsyn protein expression and nigrostriatal neurodegeneration; however, PD is a complex syndrome not only associated with dopaminergic changes, and it is clear that multiple neurotransmitters and circuitries other than the basal ganglia are also affected [2, 24]. Similarly, animal models must evolve and innovative strategies are needed to incorporate the new criteria of the neurological signs and symptoms of the disease.

Because duplication and triplication of the αsyn gene locus results in familial PD [6, 45], there continues to be substantial interest in models that overexpress αsyn to model synucleinopathies. However, targeting a large brain region or even the whole rodent brain using viral vectors has remained challenging. Recently, it was demonstrated that gene transfer throughout the CNS (central nervous system) can be achieved via intracerebroventricular injection (ICV) of adeno-associated-virus (AAV) virus into the neonatal mouse brain [39]. This method of viral vector delivery into the maturing brain allows a more efficient diffusion and infection compared to transduction of adult brain. Expression begins within days of injection and persists for the lifetime of the animal [21]. This technique provides a fast and easy means of manipulating neuronal gene expression in vivo without complex surgical intervention, or time-consuming germline transgenesis. Moreover, it has proven to be effective for modeling other neurodegenerative disorders such as frontotemporal dementia [9] and tauopathy [10]. Both of these published models developed striking behavioral and neuropathological characteristics of their respective diseases in a short period of time.

Herein, we used somatic brain transgenesis aiming to engineer and characterize a novel synucleopathy model. To this end, we expressed human wild-type αsyn by injection of AAV serotype 2/1 into lateral ventricles of postnatal day 0 (P0) non-transgenic C57BL/6 mouse pups. Histology and behavioral analysis were conducted from 1 to 6 months of age. We were able to create a novel mouse model exhibiting widespread expression of αsyn as early as 1 month post injection. Interestingly, these animals displayed pathological forms of αsyn evidenced by the presence of phosphorylated αsyn and small aggregates resistant to mild proteinase K (PK) treatment in several brain regions. However, no progressive neurodegeneration and behavioral phenotype associated with the observed pathology indicate this model may represent a pre-symptomatic stage of synucleinopathy.

## Materials and methods

### Viral vector production

The viral vector construct rAAV- αsyn and rAAV-Venus were constructed as follows: The following expression components were inserted between two AAV2 inverted terminal repeats (ITRs): The SalI**-**HindIII fragment of the pCAGGS vector (kindly provided by Mark Sands, University of Washington, St Louis) containing the hybrid CMV immediate-early enhancer/chicken β-actin promoter/exon1/intron and the poly (A) tail from rabbit beta-globin gene; full-length human wild-type αsyn cDNA(AAV-αsyn) [32]; venusYFP cDNA (AAV-venus); and the woodchuck hepatitis virus post-transcriptional regulatory element (WPRE) (kindly provided by Dr. T. J. Hope, University of Illinois at Chicago, IL, USA). Briefly, adeno-associated virus (AAV) serotype 2/1 vectors expressing full length human αsyn or venus under the control of the CMV promoter were generated by plasmid triple transfection with helper plasmids in HEK293T cells 48 h later, cells were then harvested and lysed in the presence of 0.5% sodium deoxycholate and 50 U/ml Benzonase (Sigma-Aldrich, St. Louis, MO) by freeze-thawing, and the virus was isolated using a discontinuous iodixanol gradient. The genomic titer of each virus was determined by quantitative PCR.

### Intracerebroventricular injections

All animal procedures were approved by the Mayo Clinic Institutional Animal Care and Use Committee and are in accordance with the NIH Guide for Care and Use of Laboratory Animals. Bilateral intracerebroventricular (ICV) injections were performed as previously described [5] in C57BL/6 mouse pups on postnatal day 0. Briefly, Newborn C57BL/6 mice were cryoanesthetized and subsequently placed on a cold metal plate. A 30-gauge needle was used to pierce the skull just posterior to bregma and 2 mm lateral to the midline, and 2 μl of AAV (AAV-αsyn or AAV-venus) was injected into each cerebral ventricle (1.35E + 10gc/μl). Neonatal mice were kept with parent until weaned.

Mice were sacrificed at set time points as follow: 1 month (*n* = 8 per group), 3 months (*n* = 12–15 per group) and 6 months (*n* = 12–14 per group) post-injection. Of note, 3 and 6 month groups were behaviorally assessed before being euthanized for biochemical and histological analysis.

### Behavioral analysis

At 3 and 6 months post injection mice underwent a battery of behavioral tests in collaboration with our mouse behavior core at Mayo Clinic Jacksonville. All mice were acclimated to the testing room for 1–2 h prior to testing. All behavioral equipment was cleaned with 30% ethanol prior to use with each animal. All mice were returned to their home cages and home room after each test. All behavioral tests were performed during the light cycle. To reduce experimental bias, all behavioral testing and scoring was done with the experimenter blind to the genotype of the mice.

#### Open-field assay

Mice were placed in the center of an open-field arena (40 W × 40 L × 30H cm) and allowed to roam freely for 15 min. An overhead camera was used to track movement with AnyMaze software (Stoelting Co., Wood Dale, IL), and mice were analyzed for multiple measures, including total distance traveled, average speed, time mobile, and distance traveled in an imaginary ‘center’ zone (20 × 20 cm).

#### Pole test

The pole test was performed according to Matsuura et al., 1997 [31] with minor modifications. Animals were placed head-upward on the top of a vertical wooden pole (diameter 1 cm; height 50 cm). The animals orient themselves downward and descend the length of the pole. Each mouse received 2 days of training consisting of five trials for each session. On the test day, the total time until the mouse reached the floor with its four paws was recorded (T-total) as well as the time needed for the animal to turn downward (T-turn). The best performance over the five trials was used.

#### Rotarod test

Motor coordination and balance was measured using an automated rotarod system (Med Associates, Inc). Each mouse was placed on an accelerating spindle (4–40 rpm) for 5 min for three consecutive trials with at least 20 min of rest in between trials. The latency to fall time was recorded when the mouse fell off the spindle, triggering a sensor that automatically stops the timer located underneath the spindle. The animals were trained for 3 consecutive days and test on day 4.

#### Beam traversal

This protocol is based on Southwell et al., 2009 [46]. Mice were trained for two sessions (1session/day) to walk across a beam toward their home cage (with gentle nudging, if necessary) until they were able to traverse the entire length of the beam unassisted. On the third day (test day), beam traversal was videotaped and each mouse had five trials. Videotapes were rated on slow motion by an experimenter blind to genotype and the total time taken to traverse the beam and the number of slip made during beam traversal were counted. The mean of the five trials was used as the score for each mouse.

#### Contextual and cued fear conditioning (CFC) test

This test was performed as previously described [10]. Briefly, CFC was conducted in a sound attenuating chamber with a grid floor capable of delivering an electric shock, and freezing was measured with an overhead camera and FreezeFrame software (Actimetrics, Wilmette, IL). Mice were trained and tested on 2 consecutive days. On the training day, an 80-dB white noise served as the conditioned stimulus (CS) and was presented for 30 s. During the final 2 s of this noise, mice received a mild foot shock (0.5 mA), which served as the unconditioned stimulus (US). After 1 min, another CS–US pair was presented. The mouse was removed 30 s after the second CS–US pair and returned to its home cage. Twenty-four hours later, mice were tested by being returned to the conditioning chamber for 5 min without any shock, and freezing behavior was recorded. For the auditory CS test, environmental and contextual cues were changed by: wiping testing boxes with 30% isopropyl alcohol instead of 30% ethanol; replacing white house lights with red house lights; placing a colored plastic triangular insert in the chamber to alter its shape and spatial cues; covering the wire grid floor with opaque plastic and altering the smell in the chamber with vanilla extract. The animals were placed in the apparatus for 3 min, and then the auditory CS was presented and freezing was recorded for another 3 min (cued test). Baseline freezing behavior obtained during training was subtracted from the context or cued tests to control for animal variability.

### Tissue processing

After behavioral analysis, all animals euthanized by deep anesthesia with sodium pentobarbital prior to transcardial perfusion with phosphate buffered saline (PBS). The brain was removed and bisected along the midline. Half brain was drop-fixed in 10% neutral buffered formalin (Fisher Scientific, Waltham, MA) overnight at 4 °C for histology, whereas the other half was frozen for biochemical studies. The half brain fixed in 10% formalin was embedded in paraffin wax, sectioned in a sagittal plane at 5 μm thickness and mounted on glass slides. The tissue sections were deparaffinized in xylene and rehydrated in a graded series of alcohols. Antigen retrieval was performed by steaming in distilled water for 30 min, and endogenous peroxidase activity was blocked by incubation in 0.03% hydrogen peroxide. Sections were then immunostained with anti αsyn (Covance, 4B12; BD biosciences, clone 42; Millipore, 5G4), αsyn pS129 (Wako, pSyn#64), glial fibrillar acidic protein (GFAP) (Biogenex, ARO20), ionized calcium-binding adaptor molecule 1 (iba1) (Wako, 019–19,741), and visualized using the Envision Plus system (DAKO, CA, USA). Slides were counterstained with hematoxylin, dehydrated in a graded series of alcohol and xylene, and coverslipped.To avoid undesired background staining the use of Vector MOM immunodetection KIT (Vector laboratories) was required for 4B12 and αsyn pS129 staining. According to the Kit instructions, 1 h of preincubation with blocking unspecific protein from MOM kit was followed by incubation with the primary antibody in the MOM protein concentrate at room temperature for 30 min. For double immunofluorescence sections were immunostained with primary antibody against αsyn (4B12) in combination with anti-tyrosine hydroxylase (TH; Thermos, OPA1–04050) overnight at 4 °C. For visualization fluorescent conjugated antibodies, Alexa 488-goat anti-mouse and Alexa 568-goat anti rabbit at 1:500 (Invitrogen) were used for 2 h at room temperature. For the detection of β-pleated sheets, some of the sections were incubated with 1% Thioflavin S (ThioS, Sigma) for 5 min, washed three times with 70% ethanol and two times with PBS. Sections were mounted with Vectashield mounting medium (Vector laboratories). Lastly, for proteinase K (PK) digestion, tissue sections were preincubated with proteinase K (DAKO) in PBS for 2 min at room temperature before performing regular immunostaining for αsyn using LB509 antibody (Thermo fisher).

### Western blot

Frozen hemi brains were mechanically homogenized on ice in 10% (*w*/*v*) of cold lysis RIPA buffer (Millipore) containing protease inhibitor cocktail (Roche Diagnostics), and centrifuged at 100,000 x g for 20 min. The supernatant was saved as the soluble fraction. Triton X-100 was added to the pellet (final concentration 1%) and incubated for 20 min on ice followed by centrifugation (100,000 x g for 30 min). The supernatant was designated as Triton X-100 soluble fraction. The insoluble fraction was finally dissolved in lysis buffer containing 2% SDS (sodium dodecyl sulfate) and sonicated for 10s. Protein concentration of each lysate was determined by BCA. Equal amounts of soluble and insoluble fractions were analyzed by SDS protein electrophoresis and immunoblotted for total αsyn (BD biosciences, clone 42) and actin (Sigma, A5060). Immunoreactivity was visualized using an ECL chemiluminescent detection Kit (Thermo Fisher Sciences) and images were acquired with a CCD imaging system (LAS-4000, Fujifilm, Japan).

### Taqman qRT-PCR analysis

Hemi brains were carefully dissected and snap-frozen on dry ice. Total RNA was extracted using TRIzol Reagent (Ambion Life Technology) followed by DNase RNA cleanup step using RNeasy (Qiagen, Germantown, MD). The quantity and quality of RNA samples were determined by the Agilent 2100 Bioanalyzer using an Agilent RNA 6000 Nano Chip. Complementary DNA (cDNA) synthesized from 500 ng of RNA with Applied Biosystems High-Capacity cDNA Archive Kit was used as a template for relative quantitative PCR using ABI Taqman chemistry (Applied Biosystems). mRNA expression was quantified using Hs00240906_m1 (human *SNCA)* and Mm01188700_m1 (mouse *Snca*). Mm00441941_m1 (*tfrc,* Transferrin receptor), Mm00497442 _m1 (*txnl1,* Thioredoxin-Related Protein 1) assays were used as endogenous controls for global normalization. Each sample was run in quadruple replicates on the QuantStudio 7 Real-Time PCR System (Thermo Fisher).

### Image analysis

Brightfield images were captured using the Aperio slide scanner (Vista, CA, USA). Fluorescent images were taken with a 40 x Plan-Apochromat objective using a Zeiss AxioObserver equipped with an ApoTome Imaging System (Zeiss). Microglial and astroglial cell counts and morpholological analysis (process length and cell body size) were quantified using MetaMorph Image Analysis Software (Molecular Devices) with the neurite outgrowth application module [4]. MetaMorph Software with the cell counting module was used to assess the burden of NeuN positive neurons. First, ImageScope® software (v12.1; Leica Biosystems) was used to annotate the cortex on mid-sagittal sections stained for NeuN for each mouse. Then, Positive Pixel Count Algorithm was established to recognize and quantify NeuN positive cells .The output parameter was the number of NeuN-positive neurons per given mm2 area annotated.

### Statistics

Data were analyzed using GraphPad Prism 6 (San Diego, CA) and are presented as mean ± standard error of the mean (S.E.M.). Statistical significance was determined using a Student’s t-test or one-way analysis of variance with Tukey’s multiple comparison post-hoc. *p* < 0.05 was considered significant.

## Results

### Neonatal delivery of AAV2/1-αsyn leads to widespread expression of human αsyn throughout the adult mouse brain

**Fig. 1:**
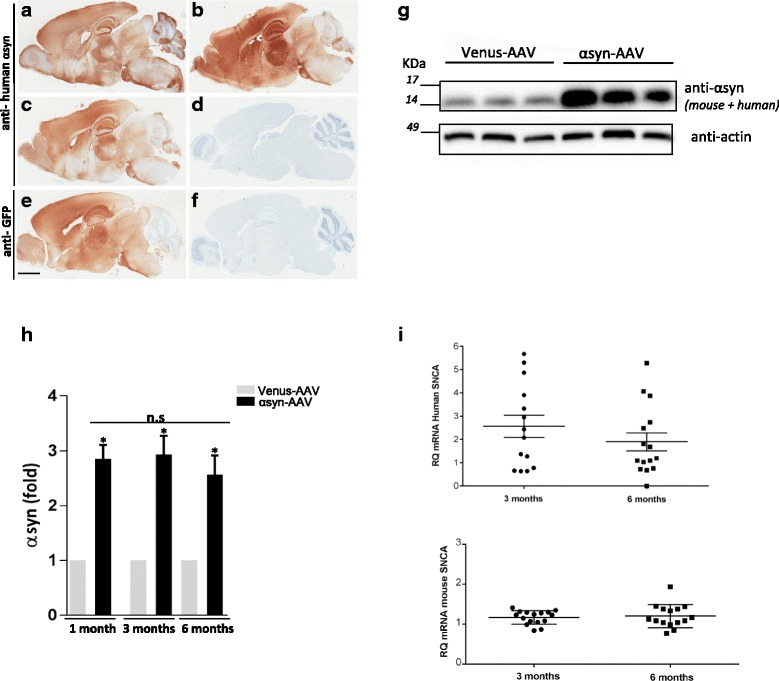
Level of expression of human wt-αsyn in AAV1 icv injected mice. **a**, **f** Photomicrographs of representative brain of animals injected with AAV1-αsyn (**a**, **b**, **c**, and **f**) and AAV-venus (**e**, **d**). Human αsyn-specific antibody reveals high level of expression throughout the brain in AAV1-syn mice at 1, 3 and 6 months after injection (**a**, **b**, **c** respectively), whereas AAV-venus injected animal is negative (**d**). When using a GFP antibody, only mice injected with the AAV-venus control show a positive staining (**e**). **g** Level of expression of the transgene was assessed by western blot at all ages. Representative animals at 3 months of age show a robust expression of total αsyn in AAV1-syn group compared to AAV-venus. Antibody recognizing human and mouse αsyn was used (clone 42). **h** Quantification of the western blot shows αsyn level increase up to 3 fold. The data are expressed as the amount of total level of αsyn normalized to actin (*, *p* < 0.05) and are from 3 repeated experiments (*n* = 8–13) per age group per genotype). **i** RT-qPCR was performed to compare the level of gene expression at 3 and 6 months of age with primers specific for human or mouse αsyn. Scale bar in e = 1 mm and apply to **a**, **b**, **c**, **d** and **f**

To assess the ability of somatic brain transgenesis of AAV2/1-αsyn at postnatal day 0 to model synucleopathy, non-transgenic animals received bilateral ICV injection of 2ul AAV1 expressing either wild-type (wt) full length human αsyn or venus as a control. Brains were harvested at 1, 3, and 6 months of age, and the level and distribution of human αsyn expression was evaluated histologically (Fig. 1a-i). The novel paradigm of neonatal viral delivery using the AAV2/1 serotype induced substantial expression of the transgene throughout the adult mouse brain as observed by immunostaining with an antibody known to be selective for human αsyn (clone 4B12) (Fig. 1a-c). Control brains injected with AAV-venus were also immunostained, and as anticipated, human αsyn burden was not detected (Fig. 1d). It is interesting to note that venus expression from the control vector closely matched that of human αsyn (Fig. 1e), suggesting a similar distribution and spread of the virus. Human αsyn expression was observed as early as 1 month post-ICV injection (Fig. 1a), and maintained at both 3 and 6 month time points (Fig. 1b and c). However, variation in labeling intensity among the animals, particularly at 6 months of age, could be observed, most probably due to small inconsistencies between the free-hand ICV injections (Additional file 1: Figure S1a). Similarly, immunoblot of half brain tissue lysates indicates sustained human αsyn expression (Fig. 1g) with levels reaching 2–3 fold over endogenous mouse αsyn expression (Fig. 1h) and analogous to level observed in well characterized transgenic αsyn mouse line, especially the line 61 [8, 43], (Additional file 1: Figure S1b, c). By 6 months of age αsyn expression levels appeared lower (not statistically significant) than the 1 and 3 month groups despite the fact that both the 3 and 6 month groups received ICV administration of AAV at the same time with animals randomly assigned to 3 and 6 month groups subsequently. To further investigated this apparent discrepancy, mRNA expression was measured on the half brain via qRT-PCR using primers specific to human or mouse αsyn. Variability was observed between animals in each group (Fig. 1i), yet no significant difference in αsyn mRNA level was detected between the 3 and 6 months groups (Fig. 1i, top). Of importance, qRT-PCR analysis revealed no significant differences in mouse αsyn levels at 3 and 6 months (Fig. 1i, bottom), suggesting that over-expression of human αsyn does not promote any regulatory effect on mRNA levels of the endogenous gene.

**Fig. 2:**
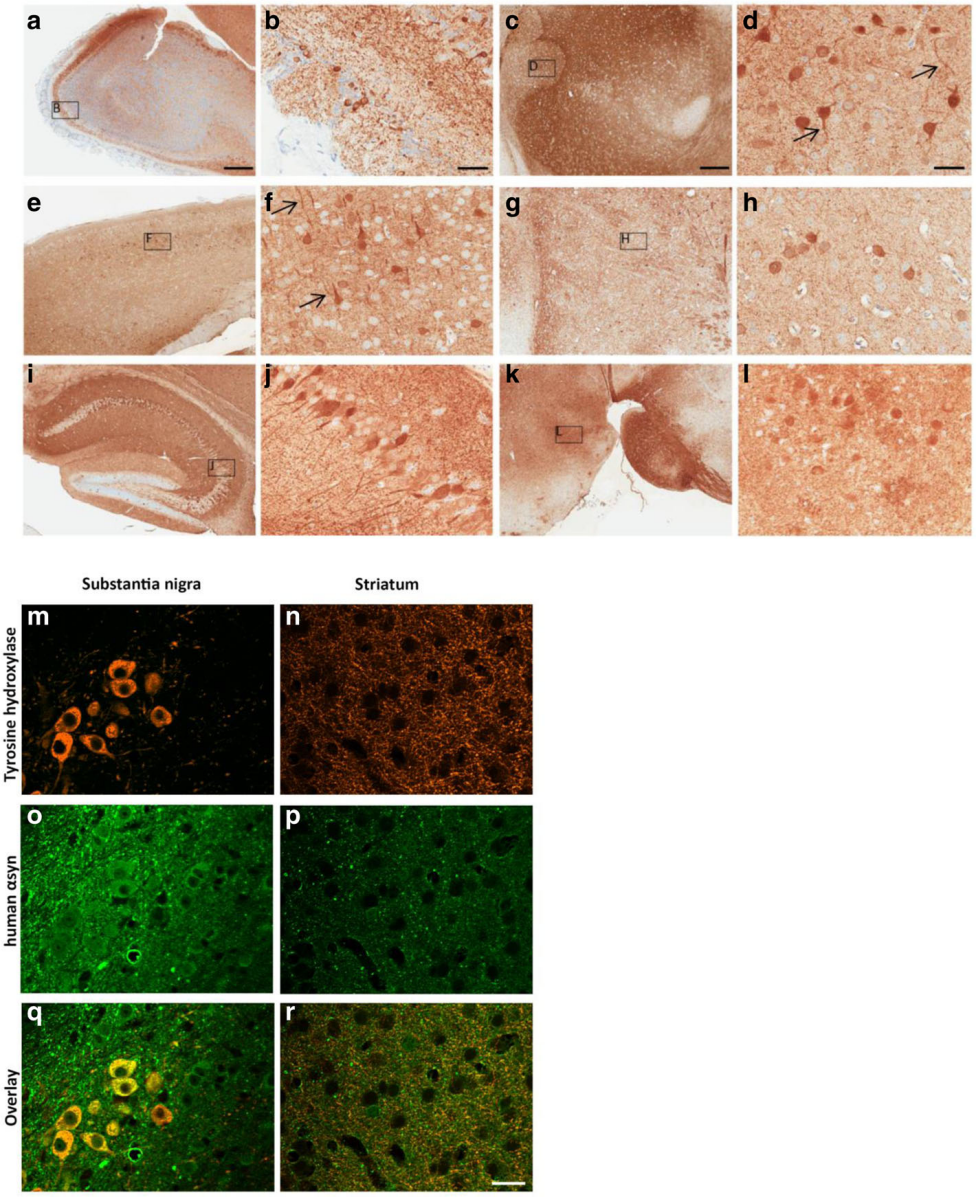
Widespread expression of the transgene throughout the whole brain of adult mice. (**a**-**l**) Photomicrographs of representative regions of 3 month old brain of AAV1-αsyn injected mice. Intense cytoplasmic staining in the olfactory bulb (**a**, **b**), thalamic (**c**, **d**) and cortical regions (**e**, **f**) with some axonal projections (*black* arrows). Also strong neuropil burden was observed in several regions within the striatum (**g**, **h**), midbrain (**k**, **l**) and hippocampus (**i**, **j**). (**m**-**r**). Co-immunostaining for human αsyn and dopaminergic (TH) neuronal marker at the level of the SN (**m**, **o**, **q**) and Striatum (**n**, **p**, **r**). TH+ cell bodies and fibers expressed the transgene as observed in overlay pictures. Scale bar in A = 500 μm and apply to **c**, **e**, **g**, **i**, **k**; Scale bar in b = 50 μm and apply to **d**, **f**, **h**, **j**, **l**; Scale bar in m = 20 μm

Histological analysis shows that the distribution pattern of human αsyn is fairly consistent between all animals included in the study, with no obvious differences between genders. Expression of the transgene is diffuse and heterogeneous from one region to another (Fig. 2a-l), and appears as soma and positive axons (Fig. 2d and f, black arrows), strong neuropil burden (Fig. 2h–l), and/or nuclear localization in some neuronal populations. These patterns were observed separately or together, depending on the area of the brain. Overall, a high level of human αsyn expression was observed in the olfactory bulb (Fig. 2a and b), thalamus (Fig. 2c and d), cortical region (Fig. 2e and f), and hippocampus (Fig. 2i and j). Also of importance for synucleinopathy models, and particularly PD, staining for human αsyn was present along the nigrostriatal pathway (Fig. 2g-l). Co-immunostaining of the dopaminergic cell population of the SN (Fig. 2m–q) and the terminals in the striatum (Fig. 2n–r) was performed using antibodies against tyrosine hydroxylase (TH) and human αsyn. At the level of the SN, in TH positive cells (Fig. 2m), cytoplasmic accumulation of αsyn was detected (Fig. 2o and q). The same was true at the level of the terminals, where TH+ fibers were immunopositive for punctate aggregates of human αsyn (Fig. 2p and r).

### Pathological markers of synucleopathy are detected in AAV2/1-αsyn transduced mouse brain.

**Fig. 3:**
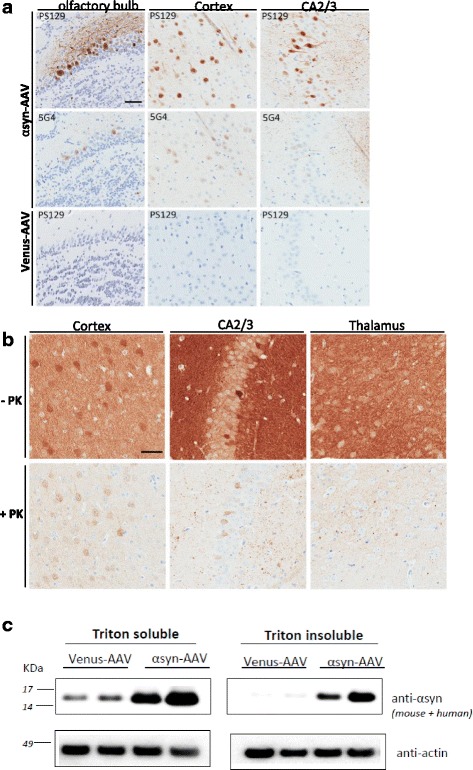
Detection of αsyn-associated pathology in AAV1-αsyn mouse. **a**, **b** Photomicrographs of representative regions of the brain of AAV1-αsyn injected mice.at 3 months of age. **a** Phosphorylated αsyn (pS129) was highly increased within the neuronal soma and to a lesser extent within the axonal projections.5G4 immunostaining was less intense but follow the same pattern as pS129. Neither pS129 nor 5G4 were found in AAV-venus animals (*bottom line*). **b** Brain sections digested with proteinase K showed PK-resistant αsyn in neuronal cell bodies and neurites with small inclusions (< 10 μm). **c** Representative Western Blot of Triton-X100 soluble and 2% SDS fraction of 3 month olds animals. Scale bars in a and b = 50 μm

In order to investigate pathological changes in AAV-αsyn-injected animals, brains were analyzed by immunohistochemistry for the presence of disease-associated αsyn immunoreactivity using antibodies specifically recognizing phosphorylated forms of αsyn (pS129) or disease-specific forms, 5G4 (Fig. 3a). Previous studies have shown that approximately 90% of αsyn accumulated in LBs in the human brain is phosphorylated at serine 129 and it is therefore considered a marker of disease-associated neuropathology [16, 36]. In the same manner 5G4 antibody has previously been shown to bind aggregated αsyn preparations and αsyn from patients with synucleinopathies, but not control cases [23]. In contrast to the total human αsyn expression described earlier (Figs. 1 and 2), disease associated αsyn burden was not diffuse but restricted to a few brain regions. The pattern was the same in all animals with no significant increase or change in cellular localization over time. Interestingly, pS129 and 5G4 burden consistently overlapped, with neurons immunopositive for 5G4 also immunopositive for pS129, although intensity of 5G4 was notably weaker (Fig. 3a, middle row). Disease-associated αsyn-positive structures consistently appeared in the olfactory bulb, cortical, and hippocampal regions (Fig. 3a, top and middle row), whereas control-injected mice were not immunopositive with any of the antibodies (Fig. 3a, bottom row). Phospho-S129 is noticeably increased within the neuronal soma, and to a lesser extent, within the axonal projections. Moderately increased phosphorylation was apparent in thalamic nuclei and the SN of some animals.

To further evaluate the nature of pathological αsyn observed, we performed immunohistochemical analyses on sections treated with proteinase K (PK), which hydrolyzes soluble proteins and retains insoluble protein aggregates. In postmortem human brains, PK resistant αsyn aggregates correlate with pathology, supporting the significance of these aggregates in synucleinopathies. In our AAV animal model, PK-resistant αsyn is evident in several brain regions at an early time point (1 month), and continued to be observed at later time points. Intraneuronal inclusions were found mostly in cortical and hippocampal regions (Fig. 3b), as observed by punctate cytoplasmic staining in the remaining αsyn positive neurons. Spherical aggregates and clumps (<10 μm) were visualized in the thalamus (Fig. 3b) and brainstem. This pattern is consistent across all ages of animals. However, these inclusions were not ThioS positive (Additional file 2: Figure S2a-i) which suggests an early aggregated form rather than β-sheet structure. Lastly, we examined the biochemical solubility of accumulate αsyn. Sequential extraction was performed using brain lysates prepared with a series of buffers with increasing strength of protein solubilization (1%Triton X-100, and 2% SDS). Insoluble aggregated αsyn was observed in the SDS soluble fraction of most of the AAV-αsyn brains at 3 and 6 months of age (Fig. 3c). In contrast, αsyn detected in the tritonX-100 fraction of AAV-venus animals is not present in the insoluble fraction.

It is noteworthy that, despite the presence of αsyn inclusions, aggregated and pS129 immunostaining, there was no apparent neurodegeneration or cell loss at 3 or 6 month. Examination of neuN immunotained sections showed no evident cell loss or degeneration of brain regions overexpressing αsyn (Additional file 2: Figure S2k-l).

### αSynuclein pathology is associated with astrogliosis with no changes in microglia profile

**Fig. 4:**
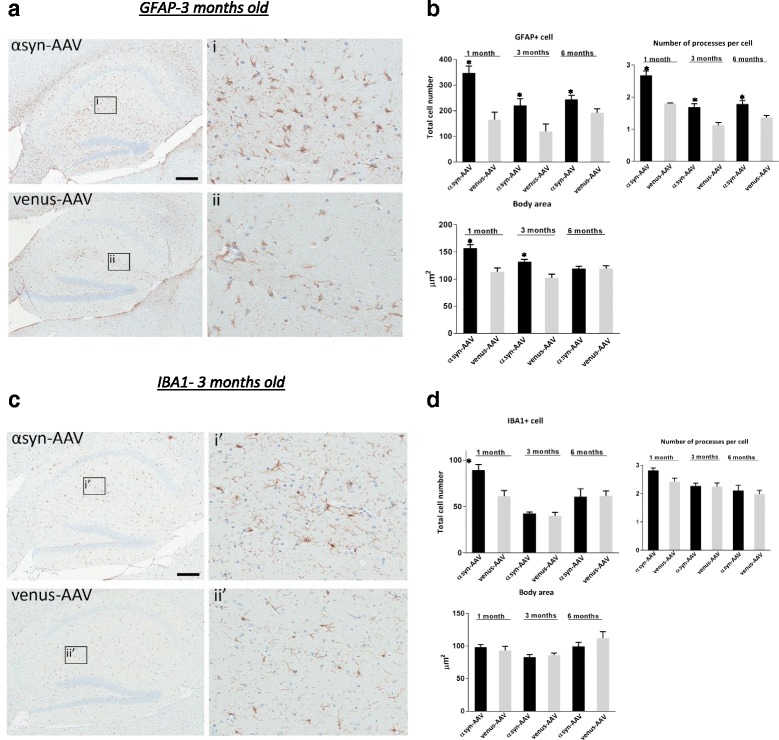
αSyn pathology is associated with astrogliosis but no changes in microglia profile. **a**, **c** Representative brain sections immunostained with anti-GFAP (**a**) and anti-iba1 (**c**) in hippocampus of 3 months old mice. **b** Detailed cell count and morphological analysis across time shows that the number of GFAP positive cells is significantly higher in AAV1-αsyn than AAV-venus, also astroglial cells present more processes and a larger body area. **d** The same morphological analysis was performed with the iba1 positive cells. At 1 months of age a higher number of microglia was observed but was not maintained at 3 or 6 months. No morphological microglia changes could be observed at 3 or 6 months of age. Scale bars in **a**, **c** = 200 μm, and i, ii, i’ and ii’ = 50 μm,* *p* < 0.001, AAV-αsyn vs AAV-venus

Several lines of evidence indicate that neuroinflammation plays an important role in the pathophysiology of PD [25]. In fact studies suggest that induction of neuroinflammation correlates with disease progression as a result of αsyn aggregation [17]. AAV-αsyn animals were immunohistologically analyzed to determine whether robust expression of αsyn results in a concomitant inflammatory response (Fig. 4). Brain sections at 1, 3, and 6 months of age were immunostained for GFAP, a marker of astrocyte activation (Fig. 4a), and iba1 a microglial marker (Fig. 4c). Increased expression of GFAP was observed in hippocampal, thalamic, and brainstem regions of αsyn transduced mice. However, the number of GFAP-positive astrocytes was significantly increased only in the hippocampus of AAV-αsyn animals at 1 month (Fig. 4b, P<0.001) and stayed significantly higher in 3 and 6 month animals (Fig. 4b) compared to age-matched AAV-venus mice. Moreover, morphological analyses highlighted a significant increase in the number of astrocytic processes over time (Fig. 4b, *P*<0.001) and larger soma areas were apparent in the 1 and 3 month cohorts (Fig. 4b, *P*<0.001). Taken together, these results suggest that αsyn overexpression is associated with astrogliosis in this rodent model. By contrast, detailed morphological analysis of microglia using anti-iba1 antibody did not show any significant difference in the number of microglia cells or in morphology (Fig. 4c and d).

### AAV-αsyn mice have no progressive PD-like motor deficits or cognitive dysfunction

**Fig. 5:**
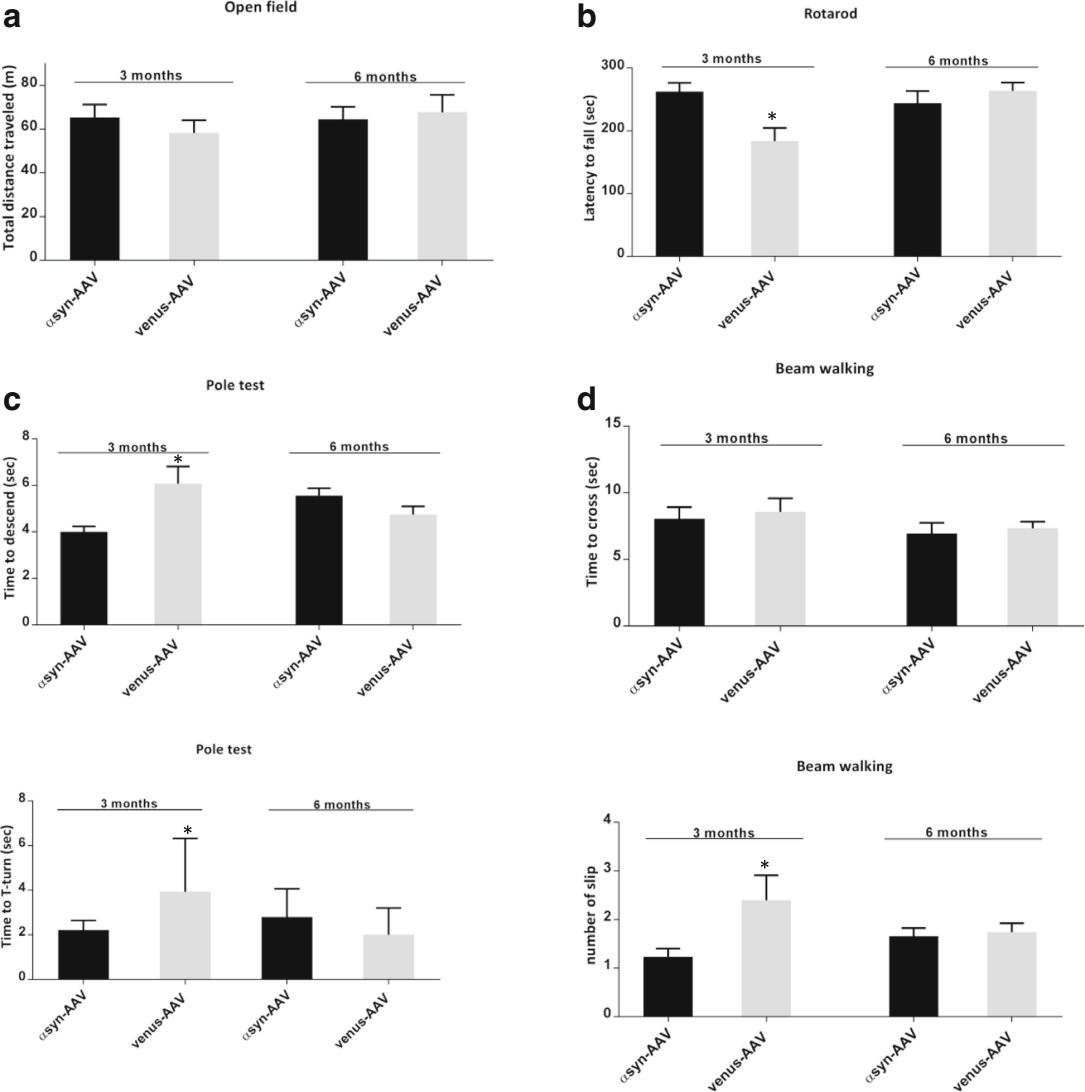
Behavioral assessment of motor functions. **a** Measurement of the total distance in the open field paradigm at 3 and 6 months revealed no change between AAV1-αsyn and AAV-venus. At 3 months of age AAV1- αsyn seem to perform better in motor coordination and balance tasks (**b**, **c**, **d**) relative to control group (AAV-venus) however, no difference at a later stage was observed. Both groups were able to stay on the accelerating rotarod (**b**) go down the pole test (**c**), and cross the beam walk without slip/errors (**d**). Data are shown as mean ± S.E.M, *n* = 12–15 per genotype/ per age, two-way ANOVA, post-hoc Bonferroni

The behavioral impact of αsyn overexpression was examined in the 3 and 6 month cohorts. We used behavioral tests designed to assess deficits in motor performance as well as cognitive performance that are characteristic of synucleopathies in humans and well characterized in some preclinical mouse models [1, 29]. AAV-αsyn mice displayed no difference in exploratory behavior in the open field task relative to control groups at 3 or 6 months as assessed by time spent in the center of the box, and time spent freezing (data not shown). Similarly, neither general locomotion activity, distance traveled (Fig 5a), nor speed (not shown), was impaired in comparison to the age matched controls. Balance and motor coordination was assessed using the accelerated rotarod task. Surprisingly this test revealed that at 3 months of age the AAV-αsyn mice stayed significantly longer on the rotating rod than the control AAV-venus (Fig. 5b, *P*<0.05 at 3 months). However, AAV-venus and AAV-αsyn mice were performing equally well at 6 months. The same trend was observed in the pole test where AAV-αsyn at 3 month seemed to perform better evidenced by the significantly shorter time taken to descend the pole (Fig. 5c, top graph *P* < 0.05). However, at 6 month of age the time to descend or turn on the pole (Fig. 5c, bottom graph) did not differ in either group. Finally the beam walk tests show no difference in time to cross at any time point (Fig. 5d, top graph). The numbers of foot slips were higher in the AAV-venus at 3 months of age (Fig. 5d, bottom graph,) but this was not the case at the latest end point time.

**Fig. 6:**
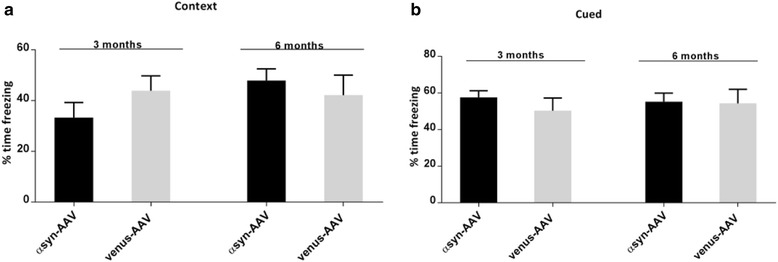
Behavioral assessment of learning and memory functions. Fear conditioning was used to measure cognitive deficits. Freezing time at 3 and 6-months old mice in the context (**a**) and cue (**b**) test was analyzed. AAV1-αsyn exhibited no significant deficits in memory and learning compared to AAV-venus. *n* = 12–15 per group

Lastly, memory impairment is often a prominent feature of LB disorders. To test whether the accumulation of abnormal αsyn affected cognitive function in these animals we performed an associative memory tests by analyzing fear conditioning behavior. We examined both contextual and cued fear memory at 3 and 6 months of age. AAV-αsyn mice did not exhibit deficits in the contextual fear conditioning paradigm, and no significant memory impairments in their ability to associate either the spatial context (Fig. 6a) or an auditory cue (Fig. 6b) could be observed compared to control.

## Discussion

Preclinical models to study αsyn-associated disease pathophysiology and to develop new therapeutic strategies are crucial tools in translational research. In the last two decades, viral vector gene delivery has offered great insights into the understanding of PD disease etiology, pathology, and molecular mechanisms. However, this method requires complex surgical intervention to locally deliver genes of interest. Herein, we generated a novel animal model, based on over-expression of human αsyn via neonatal injection, to express the transgene in the whole brain from birth to adulthood of mice. Our data demonstrate that following neonatal ICV injection, αsyn is distributed diffusely throughout the neuronal cytoplasm and also as puncta and inclusion bodies of varying size in several regions of the brain, from the most frontal part of the CNS (olfactory bulb) to the brainstem. The pathology observed in this model replicates a number of characteristics found in other in vivo models of αsyn-mediated toxicity [18, 35, 42]. Most importantly, the presence of phosphorylated αsyn and small aggregates (PK resistant) in various brain regions clearly mimics what is observed in post mortem brain of PD patients. Interestingly, no locomotor dysfunction or cognitive deficits were observed at the latest end point of our study (6 months). Despite the lack of a behavioral phenotype this model may represent an attractive tool for understanding key mechanisms taking place in the presymptomatic phases of PD and synucleopathies.

Extensive diffusion of viral particles in the brain remains a challenge in vivo. Many AAV serotypes are available, each incorporating a different viral capsid protein and mediating different transduction characteristics within the brain [38, 51]. In addition, the timing of AAV injection is a major factor that determines the overall biodistribution of the transgene. To overcome these issues, newer techniques have emerged and gene transfer throughout the CNS can now be achieved via ICV injection of AAV into the neonatal mouse brain [21, 39]. Following ICV AAV administration it is hypothesized that the virus follows the flow of the CSF through the subarachnoid space. The absence of myelinated structures in the neonate brains allows for a better diffusion of the viral particles. Several groups have recently taken advantage of neonatal ICV AAV injections to develop novel, rapid, neurodegenerative disease animal models [9, 10]. To our knowledge this is the first report of a successful transduction of human wild-type αsyn via somatic brain transgenesis. In the present study we used the serotype AAV2/1 at P0 (1–12 h postnatal) as described previously [26]. In our hand this specific serotype results in widespread expression of αsyn but the timing of injection in this case is crucial; with the expression being limited if the injection is performed after P0. Chakrabarti and colleagues [5] tested several serotypes at different time-points and reported that AAV2/8, and AAV2/9 have high biodistribution property independent of the timing of injection. Therefore a comparative study with the different serotypes may be of interest for this model. Lastly, it is worth mentioning that through our paradigm, AAV2/1 at P0, we achieved widespread transduction and pathology with a 2–3 fold increase level of total αsyn. This is very similar to level observed in well characterized transgenic αsyn mouse line, especially the line 61 [8, 43]. Importantly, it reflects the level seen in patients with SNCA gene triplication of the Swedish-American kindred [13].

Synucleinopathies including PD are a group of diseases linked by the abnormal accumulation of αsyn in various cells and brain regions, depending on the specific disease [49]. The pattern of αsyn expression and accumulation in our neonatal AAV model resembles this aspect of PD pathology. Several other pathophysiologic characteristics could also be replicated in our AAV model. Indeed, at 3 months of age we observed a change in the solubility of αsyn and could even detect small aggregates that were proteinase-K resistant. The presence of insoluble and aggregated forms of αsyn correlates with LB pathology in post mortem brains and support their importance in any preclinical model. Also, αsyn aggregates are generally found as non-fibrillar, which is in line with our observations of aggregates that were not Thioflavin S positive. Accumulation of pS129 αsyn in PD brains, as well as in animal models, suggests that this post-translational modification plays an important role in the regulation of αsyn aggregation, LB formation, and neuronal degeneration [37, 53]. Enrichment of pS129 αsyn is often observed in transgenic mouse brain [35, 42]. In our model the detection of phosphorylated αsyn was pronounced in specific brain regions, suggesting that some neurons are more vulnerable to phosphorylation events than others. Interestingly, phospho-specific immunostaining is commonly associated with the nucleus of the cell. This could be of importance as nuclear localization may further aggravate neuronal toxicity [44, 52]. In this model the presence of phosphorylated forms of αsyn do not appear to be sufficient to induce neurotoxicity as no cellular loss was observed, at least by 6 months of age. The exact implication of pS129 on αsyn aggregation and toxicity in vivo remains to be determined. Lastly, there is increasing evidence for the importance of neuroinflammation in PD pathogenesis. Our histological analysis reveals an increase of astroglial cells (GFAP) in the hippocampus of AAV-αsyn animals (Fig. 6a and b) but no change in microglia profile or number. Again, this is consistent with what is has been described in PD post mortem brain, increased dystrophic astroglia cell density [3, 11].

Traditionally, αsyn overexpressing mice develop pathology, loss of striatal dopamine, and present with locomotor dysfunction at various ages, but these cardinal features of PD are not often observed in the same lines and neurodegeneration is often small or non-existent [27, 30]. Our AAV model did not develop locomotor dysfunction at an early age (3 and 6 months), despite the presence of αsyn accumulation in the dopaminergic system. Although this is in contrast with other mice over-expressing human αsyn [15], we cannot rule out that behavioral changes may take longer to develop in this model (> 6 months) due to the neonatal administration paradigm. Also, αsyn levels are causative in PD pathogenesis and familial SNCA multiplication cases showed a dose-dependent correlation of αsyn load to the PD phenotype [45]. Even though we observed 2–3 fold increase level of total αsyn in the whole brain, we did notice a variability of expression among the animals and different degree of transduction from region to region that may explain that in our model no abnormal behavior could be observed. Recently, new rodent models has emerged using αsyn overpexression in combination with other risk factors contributing to the disease. A dual exposure of αsyn, and the pesticide, rotenone, in rodent has been reported to to precipitate motor dysfunction and nigrostriatal neurodegeneration [34]. Thus, using genetic component with environmental risk factors or other disease-causing factors may be another approach to provide relevant preclinical models that replicate motor dysfunctions.

Cognitive impairments have been rarely observed in other PD models, although less freezing behavior in the fear conditioning task was reported in the line 61 at 8 months of age [41]. As with motor functions, we may anticipate learning disabilities at a later stage. Finally, the presence of early non-motor symptoms of PD were not evaluated in the present study however future studies addressing these symptoms in the neonatal AAV transduction model would be worth addressing in future study. It will be interesting to determine if impairments in gastrointestinal function, olfaction, or sleep cycle behavior can be recapitulated.

## Conclusion

Taken together, we believe that this novel model may provide significant advantages over current transgenic models to investigate early pathogenesis. The ease of the gene delivery may offer a rapid and effective preclinical model. Although we used human wild-type αsyn in our study, the neonatal gene delivery approach described herein may be used to investigate mutated or modified forms of αsyn. Interestingly, somatic brain transgenesis may offer much more than a successful spread of the viral particles. Indeed, the possibility remains to inject two AAVs simultaneously to express multiple proteins in either overlapping or distinct neuronal populations without the constraints of complex genetic backgrounds. Furthermore one could induce αsyn overexpression in different background mice to study the interaction of αsyn and others PD related genes such as LRRK2 or even model other synucleopathies (ie DLB) given the fact that αsyn interacts with a numbers of other proteins related to neurodegeneration. For example, αsyn positive inclusions are described in tauopathies and vice versa, suggesting a co-existence of these proteinopathies [33]. Therefore somatic brain transgenesis can serve as a tool to evaluate whether the pathogenicity of αsyn can be altered in the presence of an additional insult, such as tau protein.

In conclusion, this study paves the road to a new era of preclinical models in the field of synucleinopathies .and we believe that the present model is only a premise of the possibility that somatic brain transgenesis has to offer to elucidate pathological mechanisms in PD.

## Additional files

Additional file 1: Figure S1. Representative intensity of Human αsyn immunostaining (a) Photomicrographs representative of the variability of expression observed in the different group of animals at 1, 3 and 6 months of age (b) Level of expression of the transgene was assessed by western blot in AAV-αsyn at 3 months of age and compared to transgenic mice overexpressing αsyn under Thy1 promoter (line 61) at the same age. Antibody recognizing human and mouse αsyn was used (clone 42). (c) Quantification of the western blot shows αsyn level increase of 2.93 ± 0.33 fold in the AAV-αsyn animals and 3.23 ± 0.12 fold in the line 61. .The data are expressed as the amount of total level of αsyn normalized to actin (*, *p* < 0.05) and are from 3 repeated experiments. (PDF 1678 kb)

Additional file 2: Figure S2. Neither ThioS positive structures nor neurodegeneration are observed in AAV-αsyn animals. (a-i) Sagittal brain sections were incubated with anti human asyn antibody followed by 5 min in 1% thioS solution. Thalamus (a-c) and cortex (d-f) of AAV-asyn animal show strong asyn immunoreactivity (a, d) that is not thioS- positive (b, e). As a control, human DLBD brain was co-stained in parallel. Cortical Lewy bodies positive for human asyn (g) are reactive to thioS (h, i). Representative images of NeuN-labeled cells in the cortex of AAV-asyn (*n* = 9) and AAV-venus (*n* = 7) at 6 months of age (k). Quantification of NeuN-positive cells in the whole cortex (area delineated in blue). Data are presented as as mean ± S.E.M means. Scale bars in *i* = 40 μm and applied to a-h; Scale bars in k = 2 mm. Abbreviation: DLBD; Diffuse Lewy Body Disease. (PDF 1541 kb)

## References

[1] Aarsland D, Beyer MK, Kurz MW (2008). Dementia in Parkinson's disease. Curr Opin Neurol.

[2] Bohnen NI, Albin RL (2011). The cholinergic system and Parkinson disease. Behav Brain Res.

[3] Braak H, Sastre M, Del Tredici K (2007). Development of alpha-synuclein immunoreactive astrocytes in the forebrain parallels stages of intraneuronal pathology in sporadic Parkinson's disease. Acta Neuropathol.

[4] Carrano A, Das P (2015) Altered innate immune and Glial cell responses to inflammatory stimuli in Amyloid precursor protein knockout mice. PLoS One 10. doi:ARTN e0140210. 10.1371/journal.pone.0140210 10.1371/journal.pone.0140210 PMC4598170 26447481

[5] Chakrabarty P, Rosario A, Cruz P, Siemienski Z, Ceballos-Diaz C, Crosby K, Jansen K, Borchelt DR, Kim JY, Jankowsky JL, Golde TE, Levites Y (2013). Capsid serotype and timing of injection determines AAV transduction in the neonatal mice brain. PLoS One.

[6] Chartier-Harlin MC, Kachergus J, Roumier C, Mouroux V, Douay X, Lincoln S, Levecque C, Larvor L, Andrieux J, Hulihan M, Waucquier N, Defebvre L, Amouyel P, Farrer M, Destee A (2004). Alpha-synuclein locus duplication as a cause of familial Parkinson's disease. Lancet.

[7] Chaudhuri KR, Healy DG, Schapira AH, National Institute for Clinical E (2006). Non-motor symptoms of Parkinson's disease: diagnosis and management. Lancet Neurol.

[8] Chesselet MF, Richter F (2011). Modelling of Parkinson's disease in mice. Lancet Neurol.

[9] Chew J, Gendron TF, Prudencio M, Sasaguri H, Zhang YJ, Castanedes-Casey M, Lee CW, Jansen-West K, Kurti A, Murray ME, Bieniek KF, Bauer PO, Whitelaw EC, Rousseau L, Stankowski JN, Stetler C, Daughrity LM, Perkerson EA, Desaro P, Johnston A, Overstreet K, Edbauer D, Rademakers R, Boylan KB, Dickson DW, Fryer JD, Petrucelli L (2015). Neurodegeneration. C9ORF72 repeat expansions in mice cause TDP-43 pathology, neuronal loss, and behavioral deficits. Science.

[10] Cook C, Kang SS, Carlomagno Y, Lin WL, Yue M, Kurti A, Shinohara M, Jansen-West K, Perkerson E, Castanedes-Casey M, Rousseau L, Phillips V, Bu G, Dickson DW, Petrucelli L, Fryer JD (2015). Tau deposition drives neuropathological, inflammatory and behavioral abnormalities independently of neuronal loss in a novel mouse model. Hum Mol Genet.

[11] Damier P, Hirsch EC, Zhang P, Agid Y, Javoy-Agid F (1993). Glutathione peroxidase, glial cells and Parkinson's disease. Neuroscience.

[12] Dickson DW, Fujishiro H, Orr C, DelleDonne A, Josephs KA, Frigerio R, Burnett M, Parisi JE, Klos KJ, Ahlskog JE (2009). Neuropathology of non-motor features of Parkinson disease. Parkinsonism Relat Disord.

[13] Farrer M, Kachergus J, Forno L, Lincoln S, Wang DS, Hulihan M, Maraganore D, Gwinn-Hardy K, Wszolek Z, Dickson D, Langston JW (2004). Comparison of kindreds with parkinsonism and alpha-synuclein genomic multiplications. Ann Neurol.

[14] Fernagut PO, Chesselet MF (2004). Alpha-synuclein and transgenic mouse models. Neurobiol Dis.

[15] Fleming SM, Salcedo J, Fernagut PO, Rockenstein E, Masliah E, Levine MS, Chesselet MF (2004). Early and progressive sensorimotor anomalies in mice overexpressing wild-type human alpha-synuclein. J Neurosci.

[16] Fujiwara H, Hasegawa M, Dohmae N, Kawashima A, Masliah E, Goldberg MS, Shen J, Takio K, Iwatsubo T (2002). Alpha-Synuclein is phosphorylated in synucleinopathy lesions. Nat Cell Biol.

[17] Gao HM, Kotzbauer PT, Uryu K, Leight S, Trojanowski JQ, Lee VM (2008). Neuroinflammation and oxidation/nitration of alpha-synuclein linked to dopaminergic neurodegeneration. J Neurosci.

[18] Giasson BI, Duda JE, Quinn SM, Zhang B, Trojanowski JQ, Lee VM (2002). Neuronal alpha-synucleinopathy with severe movement disorder in mice expressing A53T human alpha-synuclein. Neuron.

[19] Halliday GM, Holton JL, Revesz T, Dickson DW (2011). Neuropathology underlying clinical variability in patients with synucleinopathies. Acta Neuropathol.

[20] Ibanez P, Bonnet AM, Debarges B, Lohmann E, Tison F, Pollak P, Agid Y, Durr A, Brice A (2004). Causal relation between alpha-synuclein gene duplication and familial Parkinson's disease. Lancet.

[21] Kim JY, Ash RT, Ceballos-Diaz C, Levites Y, Golde TE, Smirnakis SM, Jankowsky JL (2013). Viral transduction of the neonatal brain delivers controllable genetic mosaicism for visualising and manipulating neuronal circuits in vivo. Eur J Neurosci.

[22] Kirik D, Rosenblad C, Burger C, Lundberg C, Johansen TE, Muzyczka N, Mandel RJ, Bjorklund A (2002) Parkinson-like neurodegeneration induced by targeted overexpression of alpha-synuclein in the nigrostriatal system. J Neurosci 22:2780-2791. doi:20026246 10.1523/JNEUROSCI.22-07-02780.2002 PMC6758323 11923443

[23] Kovacs GG, Wagner U, Dumont B, Pikkarainen M, Osman AA, Streichenberger N, Leisser I, Verchere J, Baron T, Alafuzoff I, Budka H, Perret-Liaudet A, Lachmann I (2012). An antibody with high reactivity for disease-associated alpha-synuclein reveals extensive brain pathology. Acta Neuropathol.

[24] Langston JW (2006). The Parkinson's complex: parkinsonism is just the tip of the iceberg. Ann Neurol.

[25] Lema Tome CM, Tyson T, Rey NL, Grathwohl S, Britschgi M, Brundin P (2013). Inflammation and alpha-synuclein's prion-like behavior in Parkinson's disease--is there a link?. Mol Neurobiol.

[26] Levites Y, Jansen K, Smithson LA, Dakin R, Holloway VM, Das P, Golde TE (2006). Intracranial adeno-associated virus-mediated delivery of anti-pan amyloid beta, amyloid beta40, and amyloid beta42 single-chain variable fragments attenuates plaque pathology in amyloid precursor protein mice. J Neurosci.

[27] Lo Bianco C, Ridet JL, Schneider BL, Deglon N, Aebischer P (2002). Alpha -Synucleinopathy and selective dopaminergic neuron loss in a rat lentiviral-based model of Parkinson's disease. Proc Natl Acad Sci U S A.

[28] Magen I, Chesselet MF (2010). Genetic mouse models of Parkinson's disease the state of the art. Prog Brain Res.

[29] Magen I, Chesselet MF (2011). Mouse models of cognitive deficits due to alpha-synuclein pathology. J Parkinsons Dis.

[30] Masliah E, Rockenstein E, Veinbergs I, Mallory M, Hashimoto M, Takeda A, Sagara Y, Sisk A, Mucke L (2000). Dopaminergic loss and inclusion body formation in alpha-synuclein mice: implications for neurodegenerative disorders. Science.

[31] Matsuura K, Kabuto H, Makino H, Ogawa N (1997). Pole test is a useful method for evaluating the mouse movement disorder caused by striatal dopamine depletion. J Neurosci Methods.

[32] McLean PJ, Kawamata H, Ribich S, Hyman BT (2000). Membrane association and protein conformation of alpha-synuclein in intact neurons. Effect of Parkinson's disease-linked mutations. J Biol Chem.

[33] Moussaud S, Jones DR, Moussaud-Lamodiere EL, Delenclos M, Ross OA, McLean PJ (2014). Alpha-synuclein and tau: teammates in neurodegeneration?. Mol Neurodegener.

[34] Naughton C, O'Toole D, Kirik D, Dowd E (2017). Interaction between subclinical doses of the Parkinson's disease associated gene, alpha-synuclein, and the pesticide, rotenone, precipitates motor dysfunction and nigrostriatal neurodegeneration in rats. Behav Brain Res.

[35] Neumann M, Kahle PJ, Giasson BI, Ozmen L, Borroni E, Spooren W, Muller V, Odoy S, Fujiwara H, Hasegawa M, Iwatsubo T, Trojanowski JQ, Kretzschmar HA, Haass C (2002). Misfolded proteinase K-resistant hyperphosphorylated alpha-synuclein in aged transgenic mice with locomotor deterioration and in human alpha-synucleinopathies. J Clin Invest.

[36] Nishie M, Mori F, Fujiwara H, Hasegawa M, Yoshimoto M, Iwatsubo T, Takahashi H, Wakabayashi K (2004). Accumulation of phosphorylated alpha-synuclein in the brain and peripheral ganglia of patients with multiple system atrophy. Acta Neuropathol.

[37] Oueslati A (2016). Implication of alpha-Synuclein Phosphorylation at S129 in Synucleinopathies: what have we learned in the last decade?. J Parkinsons Dis.

[38] Passini MA, Watson DJ, Vite CH, Landsburg DJ, Feigenbaum AL, Wolfe JH (2003). Intraventricular brain injection of adeno-associated virus type 1 (AAV1) in neonatal mice results in complementary patterns of neuronal transduction to AAV2 and total long-term correction of storage lesions in the brains of beta-glucuronidase-deficient mice. J Virol.

[39] Passini MA, Wolfe JH (2001). Widespread gene delivery and structure-specific patterns of expression in the brain after intraventricular injections of neonatal mice with an adeno-associated virus vector. J Virol.

[40] Polymeropoulos MH, Lavedan C, Leroy E, Ide SE, Dehejia A, Dutra A, Pike B, Root H, Rubenstein J, Boyer R, Stenroos ES, Chandrasekharappa S, Athanassiadou A, Papapetropoulos T, Johnson WG, Lazzarini AM, Duvoisin RC, Di Iorio G, Golbe LI, Nussbaum RL (1997). Mutation in the alpha-synuclein gene identified in families with Parkinson's disease. Science.

[41] Rabl R, Breitschaedel C, Flunkert S, Duller S, Amschl D, Neddens J, Niederkofler V, Rockenstein E, Masliah E, Roemer H, Hutter-Paier B (2017). Early start of progressive motor deficits in line 61 alpha-synuclein transgenic mice. BMC Neurosci.

[42] Rieker C, Dev KK, Lehnhoff K, Barbieri S, Ksiazek I, Kauffmann S, Danner S, Schell H, Boden C, Ruegg MA, Kahle PJ, van der Putten H, Shimshek DR (2011). Neuropathology in mice expressing mouse alpha-synuclein. PLoS One.

[43] Rockenstein E, Mallory M, Hashimoto M, Song D, Shults CW, Lang I, Masliah E (2002). Differential neuropathological alterations in transgenic mice expressing alpha-synuclein from the platelet-derived growth factor and thy-1 promoters. J Neurosci Res.

[44] Schell H, Hasegawa T, Neumann M, Kahle PJ (2009). Nuclear and neuritic distribution of serine-129 phosphorylated alpha-synuclein in transgenic mice. Neuroscience.

[45] Singleton AB, Farrer M, Johnson J, Singleton A, Hague S, Kachergus J, Hulihan M, Peuralinna T, Dutra A, Nussbaum R, Lincoln S, Crawley A, Hanson M, Maraganore D, Adler C, Cookson MR, Muenter M, Baptista M, Miller D, Blancato J, Hardy J, Gwinn-Hardy K (2003). Alpha-Synuclein locus triplication causes Parkinson's disease. Science.

[46] Southwell AL, Ko J, Patterson PH (2009). Intrabody gene therapy ameliorates motor, cognitive, and neuropathological symptoms in multiple mouse models of Huntington's disease. J Neurosci.

[47] Spillantini MG, Goedert M (2000). The alpha-synucleinopathies: Parkinson's disease, dementia with Lewy bodies, and multiple system atrophy. Ann N Y Acad Sci.

[48] Spillantini MG, Schmidt ML, Lee VM, Trojanowski JQ, Jakes R, Goedert M (1997). Alpha-synuclein in Lewy bodies. Nature.

[49] Trojanowski JQ, Lee VM (2003). Parkinson's disease and related alpha-synucleinopathies are brain amyloidoses. Ann N Y Acad Sci.

[50] Ulusoy A, Decressac M, Kirik D, Bjorklund A (2010). Viral vector-mediated overexpression of alpha-synuclein as a progressive model of Parkinson's disease. Prog Brain Res.

[51] Vite CH, Passini MA, Haskins ME, Wolfe JH (2003). Adeno-associated virus vector-mediated transduction in the cat brain. Gene Ther.

[52] Wakamatsu M, Ishii A, Ukai Y, Sakagami J, Iwata S, Ono M, Matsumoto K, Nakamura A, Tada N, Kobayashi K, Iwatsubo T, Yoshimoto M (2007). Accumulation of phosphorylated alpha-synuclein in dopaminergic neurons of transgenic mice that express human alpha-synuclein. J Neurosci Res.

[53] Wang Y, Shi M, Chung KA, Zabetian CP, Leverenz JB, Berg D, Srulijes K, Trojanowski JQ, Lee VM, Siderowf AD, Hurtig H, Litvan I, Schiess MC, Peskind ER, Masuda M, Hasegawa M, Lin X, Pan C, Galasko D, Goldstein DS, Jensen PH, Yang H, Cain KC, Zhang J (2012). Phosphorylated alpha-synuclein in Parkinson's disease. Sci Transl Med.

