# Supplementary figures and images for: Neonatal AAV delivery of alpha-synuclein induces pathology in the adult mouse brain
Source: Acta Neuropathol Commun. 2017 Jun 23;5:51. doi: 10.1186/s40478-017-0455-3 (PMC5481919; doi:10.1186/s40478-017-0455-3)

Figure S1.

A

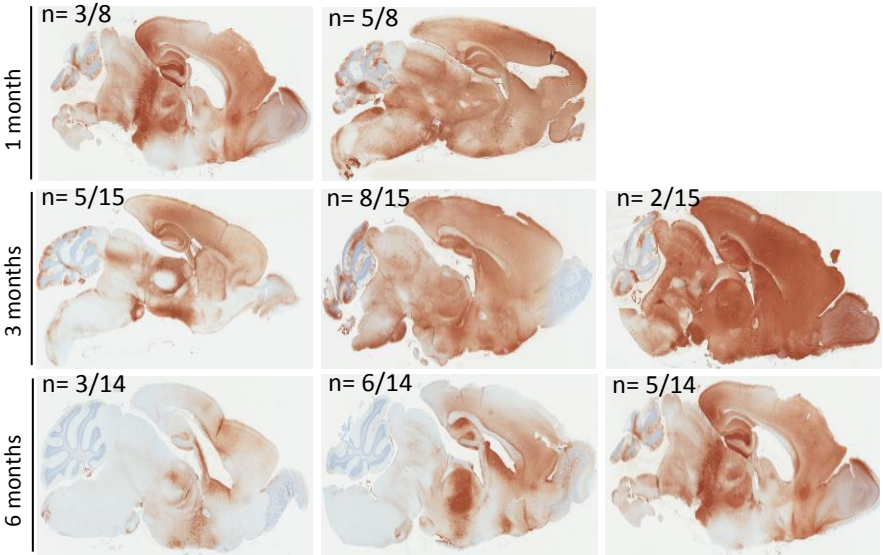

B

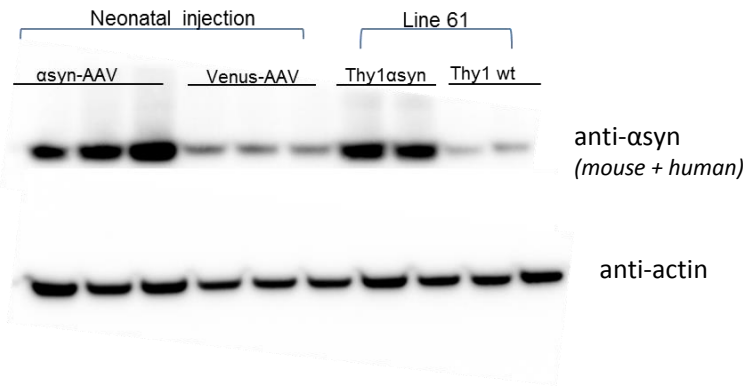

C

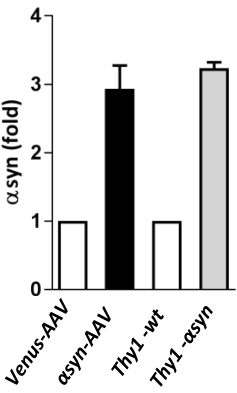

Supplement: Supplementary file 1 — Representative intensity of Human αsyn immunostaining (a) Photomicrographs representative of the variability of expression observed in the different group of animals at 1, 3 and 6 months of age (b) Level of expression of the transgene was assessed by western blot in AAV-αsyn at 3 months of age and compared to transgenic mice overexpressing αsyn under Thy1 promoter (line 61) at the same age. Antibody recognizing human and mouse αsyn was used (clone 42). (c) Quantification of the western blot shows αsyn level increase of 2.93 ± 0.33 fold in the AAV-αsyn animals and 3.23 ± 0.12 fold in the line 61. .The data are expressed as the amount of total level of αsyn normalized to actin (*, p < 0.05) and are from 3 repeated experiments. (PDF 1678 kb) [file 40478_2017_455_MOESM1_ESM.pdf]

Figure S2.

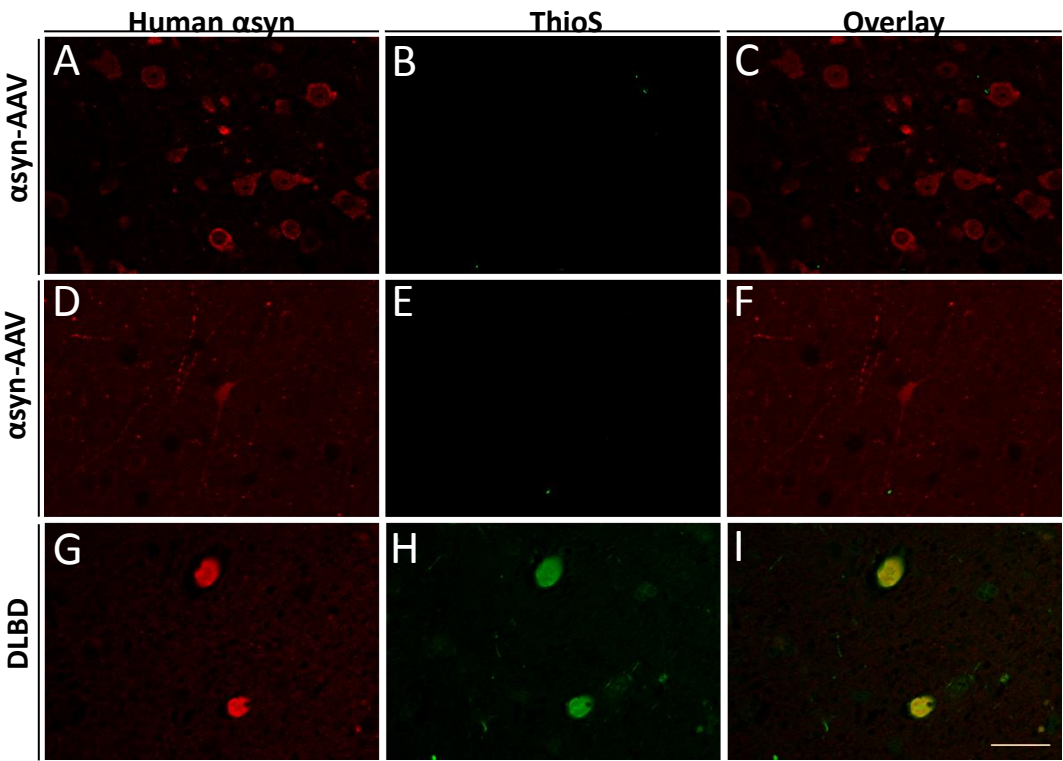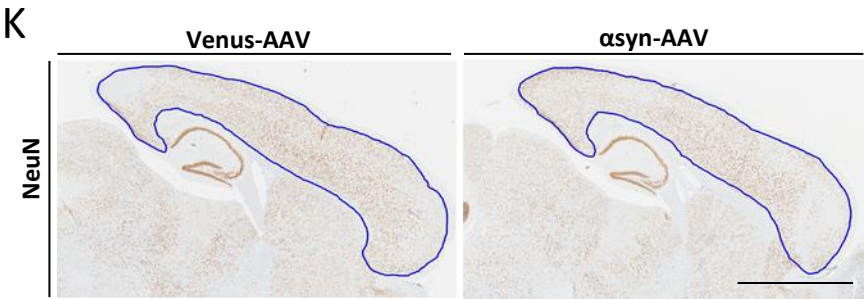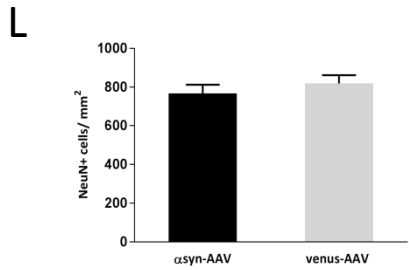

Supplement: Supplementary file 2 — Neither ThioS positive structures nor neurodegeneration are observed in AAV-αsyn animals. (a-i) Sagittal brain sections were incubated with anti human asyn antibody followed by 5 min in 1% thioS solution. Thalamus (a-c) and cortex (d-f) of AAV-asyn animal show strong asyn immunoreactivity (a, d) that is not thioS- positive (b, e). As a control, human DLBD brain was co-stained in parallel. Cortical Lewy bodies positive for human asyn (g) are reactive to thioS (h, i). Representative images of NeuN-labeled cells in the cortex of AAV-asyn (n = 9) and AAV-venus (n = 7) at 6 months of age (k). Quantification of NeuN-positive cells in the whole cortex (area delineated in blue). Data are presented as as mean ± S.E.M means. Scale bars in i = 40 μm and applied to a-h; Scale bars in k = 2 mm. Abbreviation: DLBD; Diffuse Lewy Body Disease. (PDF 1541 kb) [file 40478_2017_455_MOESM2_ESM.pdf]
